# How Far Are We from the Completion of the Human Protein Interactome Reconstruction?

**DOI:** 10.3390/biom12010140

**Published:** 2022-01-15

**Authors:** Georgios N. Dimitrakopoulos, Maria I. Klapa, Nicholas K. Moschonas

**Affiliations:** 1 Laboratory of General Biology, School of Medicine, University of Patras, 26500 Patras, Greece; geodimitrak@upatras.gr; 2 Metabolic Engineering and Systems Biology Laboratory, Institute of Chemical Engineering Sciences, Foundation for Research and Technology Hellas (FORTH/ICE-HT), 26504 Patras, Greece; mklapa@iceht.forth.gr

**Keywords:** protein–protein interactions, human PPI network, databases, network analysis, graph clustering

## Abstract

After more than fifteen years from the first high-throughput experiments for human protein–protein interaction (PPI) detection, we are still wondering how close the completion of the genome-scale human PPI network reconstruction is, what needs to be further explored and whether the biological insights gained from the holistic investigation of the current network are valid and useful. The unique structure of PICKLE, a meta-database of the human experimentally determined direct PPI network developed by our group, presently covering ~80% of the UniProtKB/Swiss-Prot reviewed human complete proteome, enables the evaluation of the interactome expansion by comparing the successive PICKLE releases since 2013. We observe a gradual overall increase of 39%, 182%, and 67% in protein nodes, PPIs, and supporting references, respectively. Our results indicate that, in recent years, (a) the PPI addition rate has decreased, (b) the new PPIs are largely determined by high-throughput experiments and mainly concern existing protein nodes and (c), as we had predicted earlier, most of the newly added protein nodes have a low degree. These observations, combined with a largely overlapping k-core between PICKLE releases and a network density increase, imply that an almost complete picture of a structurally defined network has been reached. The comparative unsupervised application of two clustering algorithms indicated that exploring the full interactome topology can reveal the protein neighborhoods involved in closely related biological processes as transcriptional regulation, cell signaling and multiprotein complexes such as the connexon complex associated with cancers. A well-reconstructed human protein interactome is a powerful tool in network biology and medicine research forming the basis for multi-omic and dynamic analyses.

## 1. Introduction

The analysis of the genetic architecture of diseases and pathophysiologies based on the structure and regulation of biomolecular networks evolved significantly after 2005, when the first extensive reconstructions of the human protein interactome based on high-throughput experiments appeared in the literature [1,2]. These advances gave rise to the field of Network Medicine, in which graph theory analysis has been applied to study the human protein interactome to further our understanding of the molecular mechanisms of diseases and cellular function [3,4,5,6]. Thus, efforts to curate all the published human protein interactions into large primary collections increased and, except from human-specific source databases such as HPRD (Human Protein Reference Database) [7], extensive datasets have been included in multi-species repositories, such as BioGRID (Biological General Repository for Interaction Datasets) [8] and IntAct (Molecular Interaction Database) [9]. These human primary protein–protein interaction (PPI) datasets have evolved through new advanced experiments over the years, but their limited overlap has been repetitively indicated [10,11,12]. In light of this issue, meta-databases, which integrate primary datasets from multiple resources, have been developed (ConsensusPathDB [13], HIPPIE (Human Integrated Protein-Protein Interaction rEference) [14], iRefIndex (Interaction Reference Index) [15], APID (Agile Protein Interactomes DataServer) [16], MatrixDB (The Extracellular Matrix Interaction Database) [17] and PICKLE (Protein InteraCtion KnowLedgebasE) [11,12,18]). These meta-datasets evolve along with the source databases and the human genome annotation, and despite their differences in primary PPI dataset integration, they are considered to be providing the full currently known human protein interactome. However, sixteen years after the first experimentally supported high-throughput instance of the human protein interactome, the scientific community is still wondering how close the completion of the genome-scale human PPI network reconstruction is and what needs to be further explored and/or corrected, in relation to current issues in the annotation of certain parts of the human genome and transcriptome. These questions need answers as we need to know whether the biological insights that we gain from the holistic investigation of the human protein interactome are valid, despite the incompleteness of the current reconstruction.

PICKLE meta-database, developed by our group [11,12,18], has unique characteristics over other existing PPI meta-databases, enabling the evaluation of the expansion of the human protein interactome and the involved primary PPI datasets through the comparison of consecutive PICKLE releases since its initial collection in 2013 [11]. More specifically, the PICKLE meta-database integrates the primary PPI datasets over the genetic information ontological network of the manually curatedUniProt/SwissProt reviewed human complete proteome (RHCP), which is used as the reference protein set. RHCP is supported by the proteomics data of NeXtProt, the reference knowledge base for the Human Protein Organization (HUPO) human proteome project (HPP) [19,20]. In this way, a comparison between different PICKLE releases can provide information about the global (over the entire proteome) and the local (around specific nodes) expansion of the human protein interactome at any level of genetic information (gene, RNA or protein), also providing information about the part of the proteome that remains without known PPIs [11,12].

In this context, we used regularly updated releases of PICKLE to evaluate the expansion of the experimentally detected human protein interactome and argue on the extent of its completeness. To this end, we performed network analysis and evaluated the evolvement of the hubs, the k-core—the densest part of the network—and the newly added nodes and edges. Furthermore, we applied two clustering algorithms to investigate the topology of the human protein interactome and to what extent certain revealed protein neighborhoods coincide with biological processes and/or multiprotein complexes. Our results regarding the evolution of the human proteome with and without PPIs is also discussed in the context of the progress of NeXtProt over the last decade with respect to the experimental evidence level of the human proteins. NeXtProt has undertaken the efforts for the validation of the protein products of human genes through integrated analysis of proteomic, localization, imaging and PPI data [21,22,23].

## 2. Materials and Methods

### 2.1. The PICKLE PPI Meta-Database

PICKLE (www.pickle.gr) [11,12,18] is a publicly available meta-database for the human and mouse direct PPI networks. In the human reconstruction, it integrates three major source PPI databases (BioGRID, reporting PPIs at the gene ID level [8]; IntAct, reporting PPIs at the UniProt ID level [9]; and HPRD, reporting PPIs at the nucleotide (mRNA) level [7]); initial releases had used the independent datasets of MINT (Molecular INTeraction database) [24] and DIP (Database of Interacting Proteins) [25], reporting PPIs at the UniProt ID level. PICKLE collects the primary PPI datasets at the genetic information level at which they are stored in each source database. Then, the primary PPI dataset integration is based on the genetic information ontology network of RHCP, excluding the *a priori* normalization to a pre-selected level of genetic information. This unique feature enables the consistent and comparable reconstruction of the human protein interactome at both the gene and the protein levels. In PICKLE 2.0, a systematic evaluation scheme was established, scoring the reliability of a PPI being direct based on the supporting experimental evidence [12]. Based on this score, the human direct PPI network is reconstructed at the following three filtering modes: unfiltered, standard and default (cross-checked)—strictest. The unfiltered dataset contains all collected PPIs; the standard dataset filters out from each primary dataset the PPIs considered of low reliability of being direct based on the experimental methods used for their detection, as the latter are reported by the source database. Finally, the strictest cross-checked (default) dataset retains the PPIs of high reliability of being direct after the primary PPI dataset cross-checking, which revises the reliability score of a PPI being direct by combining the experimental evidence from all source databases reporting this PPI. In this study, we compared the default human PPI network reconstructions at the UniProt level of PICKLE releases 1.0, 2.1, 2.2, 2.3, 2.4, 2.5, 2.6 and 3.2. It is noted that the PICKLE 1.0 dataset corresponds to the standard filtering level for PICKLE after version 2.0.

### 2.2. Network Analysis and Visualization—Protein Functional Analysis

Network analysis and metric identification including k-core [26], was performed using the relevant Cytoscape software v. 3.7.1 plugins [27]. The discussed PPI networks were visualized using Cytoscape software v. 3.7.1 or the Cytoscape visualization module of the PICKLE website. The biological role of protein groups of interest, e.g., the proteins with no known PPIs, was investigated using DAVID (Database for Annotation, Visualization, and Integrated Discovery) functional analysis [22,23].

### 2.3. Clustering Analysis

Clustering analysis of the PPI human protein interactome to detect densely connected neighborhoods was based on two different methods, Random Walk (RW) algorithm [28] and N2V-HC algorithm [29]. Each algorithm provides a different perspective of the neighborhood architecture in the human protein interactome. The former is based on the notion that if two nodes belong to the same community, there is a higher probability of reaching the second node starting from the first within a few steps. In this context, the algorithm performs a large number of short walks, by starting randomly from a node and moving to a connected node (with uniform probability over the neighbors) up to a predefined number of steps. Hence, the distances between nodes are estimated based on the probability that two nodes are visited during the same random walk. Finally, the algorithm performs a hierarchical agglomerative clustering. The iGraph package in R programming language was used [30]. The N2V-HC algorithm employs a novel approach, first constructing a network embedding from network topology and then employing hierarchical clustering to extract clusters. Briefly, given a network with N nodes, the N2V-HC algorithm builds a vector representation of dimension D << N for each node, using random walks to determine the node neighborhood. Hence, network data are converted to a numerical array (dimensionality of NxD), which can be used directly as an input to any machine learning algorithm. Hierarchical agglomerative clustering with an average linkage and Euclidean distance is applied. Then, the dynamic tree-cut method is used to split the dendrogram into clusters.

In this study, we opted to discuss, as more biologically insightful, the largest intersections between clusters of both algorithms. The ‘number of steps’ parameter of RW was set to 4, while for N2V-HC, the parameter values were set to the same values as in [29], i.e., D to 128, walk length to 80, number of walks to 10, window size to 10 and minimum cluster size to 20. These parameter sets lead to clusters of similar stringency level, i.e., similar distribution of sizes, for both algorithms.

## 3. Results

### 3.1. The Expansion of the Direct Human PPI Network

**Figure 1:**
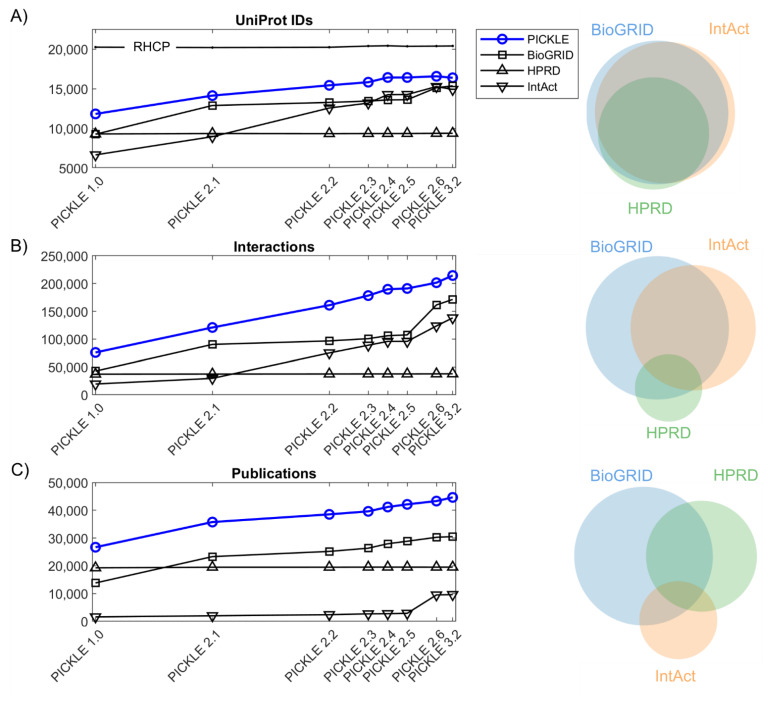
The expansion of the human protein interactome in terms of (**A**) proteins; (**B**) protein interactions; (**C**) supporting publications, based on PICKLE successive releases. The corresponding expansion of the primary PPI datasets integrated in PICKLE is also shown. The Venn diagrams on the right depict the relevant overlap between the primary datasets in PICKLE 3.2. It is noted that since PICKLE 2.6, the meta-database integrates the full IntAct dataset, while in the previous versions only the IntAct and MINT-annotated PPIs were incorporated from IntAct; DIP was integrated as a separate set from its original resource.

The experimentally determined direct PPI network in humans in PICKLE 3.2 comprises 214,446 PPIs between 16,384 UniProt IDs, supported by 44,634 publications. Since PICKLE 1.0, there has been a gradual total increase of 38.5% in UniProt IDs, 182.3% in PPIs and 67.2% in supporting publications (Figure 1). The rate of expansion has decreased in recent years in all the markers, UniProt IDs (mainly), PPIs and supporting references. Eighty per cent of the RHCP proteins have been included in the default (cross-checked) human PPI network reconstruction in PICKLE 3.2, with this number increasing to ~88% in the unfiltered PPI dataset, while it was ~60% in PICKLE 1.0. New experiments enrich the human protein interactome, mainly with new PPIs of already existing nodes, as the rate of protein node addition is four-fold smaller than that of PPI edges. Moreover, the increase in the experimentally supported PPIs is about two-fold larger than the respective change in the number of supporting references, implying that lately, PPIs are largely determined by high-throughput experiments (Supplementary Figure S1). Specifically, the average number of PPIs per publication has increased from 2.8 in PICKLE 1.0 to 4.8 in PICKLE 3.2, the number of high-throughput studies contributing more than 1000 interactions has doubled (23 in PICKLE 3.2 compared to 12 in PICKLE 2.1), and the studies with over 100 interactions increased to 175 in PICKLE 3.2 from 121 in PICKLE 2.1. Despite the extensive research in human PPIs, in PICKLE 3.2, 79% of the PPIs are still supported by a single publication only, a small decrement compared to PICKLE 2.1 (84%). These findings indicate that validation experiments are still needed for a large number of the determined PPIs.

It is noted that, in all the releases, the PICKLE human PPI network steadily includes at least ~25% more PPIs compared to the largest incorporated primary PPI dataset offered by BioGRID. This observation underlines the need for the integration of source databases for the reliable reconstruction of the currently known human protein interactome, due to their relatively limited overlap (Figure 1, Supplementary Table S1). We underline though that as the protein interactome expands to include information about different sets of interactions between the various isoforms of a protein [31], their curation at the gene level (as in BioGRID) may lead to biases in certain neighborhoods of the network as the interactions of all the isoforms are attributed to one gene and the higher biological resolution is missed.

### 3.2. Proteins without Experimentally Detected Interactions

The PICKLE reconstruction of the human PPI network is based on the RHCP as the reference protein set. The RHCP is largely the same in all the PICKLE instances since 2013 (less than 1% average difference among versions) (Supplementary Table S2). This feature allows us to follow the local expansion of the human PPI network around specific protein nodes or protein neighborhoods that are of interest. In addition, we can determine the set of RHCP proteins that have no experimentally detected interactions and determine its evolution. We note that RHCP contains UniProt IDs of all the protein evidence (PE) levels (from PE1: evidence at protein level to PE5: uncertain), which explains the constancy in the RHCP size over the last decade. The HUPO HPP is constantly updating the PE1 group by upgrading protein products from the other PE levels based on existing evidence. Supplementary Table S3 includes a comparison of the UniProt/SwissProt RHCP datasets used in the various PICKLE releases with chronologically relevant NeXtProt datasets with respect to their PE1-PE5 protein compositions. In PICKLE 1.0, ~40% of the RHCP proteins were without PPIs, while in PICKLE 3.2, 12% of the RHCP proteins still remain without experimentally supported PPIs (Supplementary Figure S2 and Table S4), taking into consideration the unfiltered human PPI network of PICKLE. Supplementary Table S4A shows the PE level of the proteins without PPIs in all the PICKLE releases based on UniProt/SwissProt and NeXtProt, while Supplementary Table S4B indicates their cumulative composition in PE1-PE5 protein products. When comparing Supplementary Tables S3 and S4B, it becomes apparent that ~80% of the uncertain (PE5) proteins in PICKLE 3.2 are without PPIs. However, they constitute only ~20% of the protein group without PPIs. On the other hand, a considerable fraction (53%) of proteins without PPIs belongs to the PE1 and PE2 (evidence at transcript level) groups. The fact that these proteins are still without identified PPIs suggests that either they are not expected to have any based on their biological role—a hypothesis in need of further investigation, or their PPIs have to be searched for by targeted experiments. Indeed, the DAVID functional annotation clustering analysis identified a large sub-group comprised of proteins related with GO:0004984 “olfactory receptor activity”, GO:0004930 “G-protein coupled receptor activity” and GO:0004888 “transmembrane signaling receptor activity”. These proteins have specialized functions and are expressed in specific tissues, which might be the reason that generic high-throughput experiments cannot provide information about their interactions. Targeted experiments are required to extend our knowledge regarding the PPIs of these proteins. Cumulatively, these results show that most of the robustly defined RHCP has already been included in the human protein interactome. However, a further investigation for any PPIs of the proteome fraction without PPIs is still required. Knowing which proteins belong to this group can substantially contribute to this effort and PICKLE provides this information.

### 3.3. Network Analysis

A comparison of network metrics between the various PICKLE releases (Supplementary Table S5) indicated that the connectivity of the human protein interactome has increased. This may be expected due to the addition of many new PPIs on already existing protein nodes, which tend to connect protein neighborhoods that had been further apart in previous releases. Specifically, we observed a decrease in the clustering coefficient, the network diameter and the characteristic path length of the network. As the clustering coefficient reflects the tendency of the neighbors of a node to form a fully connected network, and the network and characteristic path length show the number of edges in the longest and average shortest path of the network, respectively, our observations show that remote nodes tend to become more connected with the rest of the network. Moreover, the number of connected components has been largely reduced, with most of the small, isolated components consisting of up to four nodes that existed in PICKLE 1.0 and 2.1 becoming connected via some new PPIs to the largest connected component containing the vast majority of the nodes.

**Figure 2:**
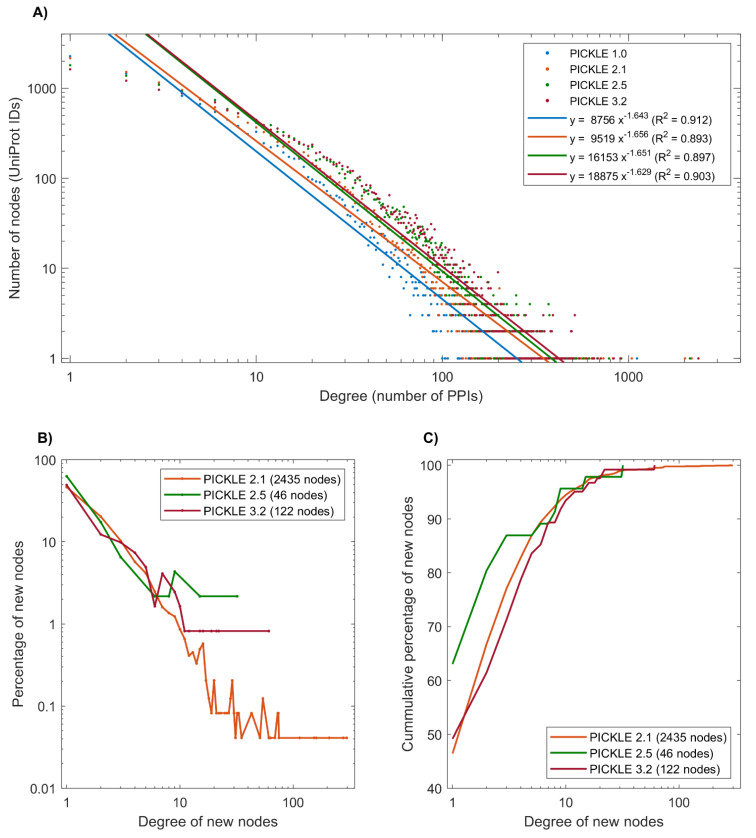
Network analysis of the human protein interactome. (**A**) The degree distribution of the network throughout PICKLE releases, following the power law. (**B**) The degree distribution and (**C**) The cumulative degree distribution for the newly inserted proteins in representative PICKLE releases. The majority of new nodes have a degree lower than 5, while none are considered as a hub, since the maximum degree is less than 300.

The human protein interaction network follows the power law; however, we do not see any change in the R^2^ fit score, which remains ~90% for all versions (Figure 2A). It is noteworthy that the nodes with a low degree become enriched with newly inserted edges, explaining the reduction in the total number of nodes having less than five edges in newer PICKLE versions (Supplementary Table S6A). Supplementary Table S6A also shows the PE level of each protein in the consecutive PICKLE releases based on UniProt/SwissProt. Supplementary Table S6B shows the composition of each PICKLE PPI dataset in PE1–PE5 proteins. Only a small fraction (less than 1%) of the node-set of the human PPI network refers to PE5 proteins, all with a degree lower than 26 in PICKLE 3.2. This observation further supports the validity of the network reconstruction, as the contribution of the uncertain proteins is considerably small; their exclusion is not expected to largely affect the insights gained from network analysis. On the other hand, further investigation of the validity of these protein products and their PPIs is required.

Among the 122 newly added proteins in the human protein interactome in PICKLE 3.2, 96 have 1–4 interactions, while none have been identified among the hubs of the network, i.e., nodes with more than 300 interactions (Figure 2B). Similarly, in other PICKLE versions, over 80% of the newly inserted nodes had a degree up to four, while 95% of them had a degree up to 12 (Figure 2C). This observation agrees with our earlier prediction, that the majority of the newly added proteins will have a low number of connections and that the human protein interactome structure regarding its hubs had largely been defined since the inaugural PICKLE release in 2013 [11]. Any hubs that have not been identified from high-throughput experiments thus far, are expected to be either spatial- or condition-specific, requiring targeted specialized experiments. For example, huntingtin, the Huntington disease protein, was introduced as a new hub of the network in PICKLE 2.6 due to a targeted PPI experiment [32].

**Figure 3:**
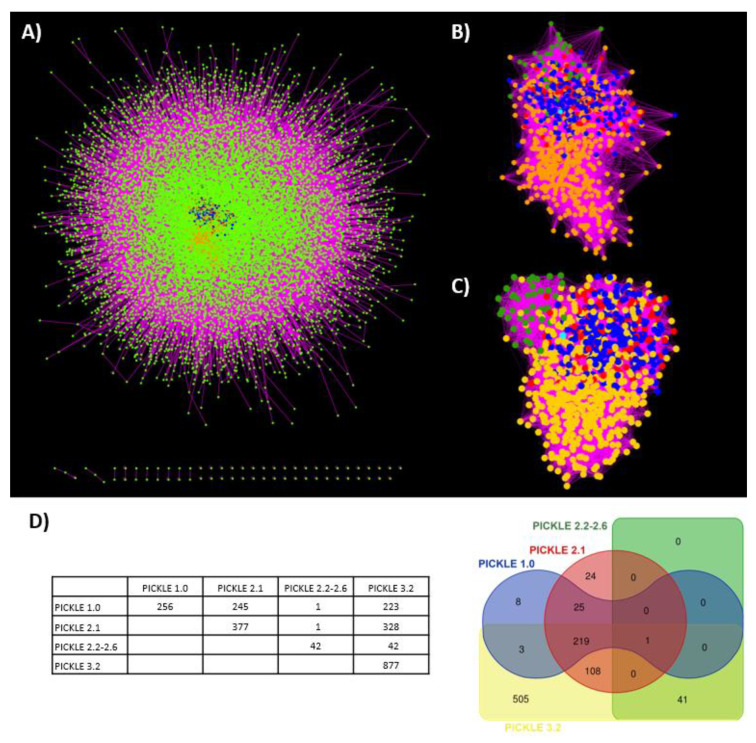
The human protein interactome of PICKLE 3.2 and its k-core in relation to the k-cores of previous PICKLE releases. (**A**) The position of the k-core in the PICKLE 3.2 network; (**B**) The k-core as extracted from the full network and (**C**) the k-core in force-directed layout; (**D**) the correlation matrix and the Venn diagram of the k-cores of the various PICKLE releases. Blue, red and green nodes depict, respectively, the proteins in the k-core intersection of PICKLE 3.2 with PICKLE 1.0 (222 proteins) or PICKLE 2.1 (108 proteins; the 219 proteins common with PICKLE 1.0 are depicted in blue) or PICKLE 2.2-2.6 (41 proteins). The unique PICKLE 3.2 k-core protein-nodes are colored yellow. The common protein in all the k-cores, P56524–HDAC1_HUMAN, is depicted in cyan.

Finally, estimation of the k-core of the human PPI network throughout its evolution indicated that the PICKLE 3.2 k-core includes both the k-core of PICKLE 1.0 and 2.1 and the apparently different k-core observed in the PICKLE 2.2–2.6 releases (Figure 3). The k-core represents the most connected protein neighborhood of the graph. Figure 3A shows the central position of the k-core of PICKLE 1.0 (blue dots), PICKLE 2.1 (red dots), PICKLE 2.2–2.6 (green dots) and PICKLE 3.2 (yellow dots) in the full human protein interactome of PICKLE 3.2. Figure 3B shows only the k-core proteins, having hidden the rest of the interactome nodes. Figure 3C shows the same sub-network in a force-directed format; the relative position of the four different k-cores is more apparent, indicating the difference between the PICKLE 2.2–2.6 k-core and those of PICKLE 1.0 and 2.1. However, all belong to the k-core of PICKLE 3.2. This observation further supports the increase in the connectivity of the human protein interaction network since its earliest instances, as the practically related protein neighborhoods appearing as rather distinct k-cores between PICKLE 2.2-2.6 and the previous releases got connected with the addition of new PPIs in PICKLE 3.2. Moreover, this result implies a rather well-defined representation of the human protein network even in PICKLE earlier instances. Interestingly, histone deacetylase 4 (P56524–HDAC1_HUMAN) is the only protein common in all the k-cores. This protein is involved in the deacetylation of lysine residues of the core histones (H2A, H2B, H3 and H4). Among other functions, it is involved in the MTA1-mediated epigenetic regulation of ESR1 expression in breast cancer [33]. Chromosome 2q37.3 microdeletions encompassing HDAC4 and the point mutations of this gene have been associated with the brachydactyly mental retardation syndrome [34,35].

### 3.4. Clustering Analysis

**Figure 4:**
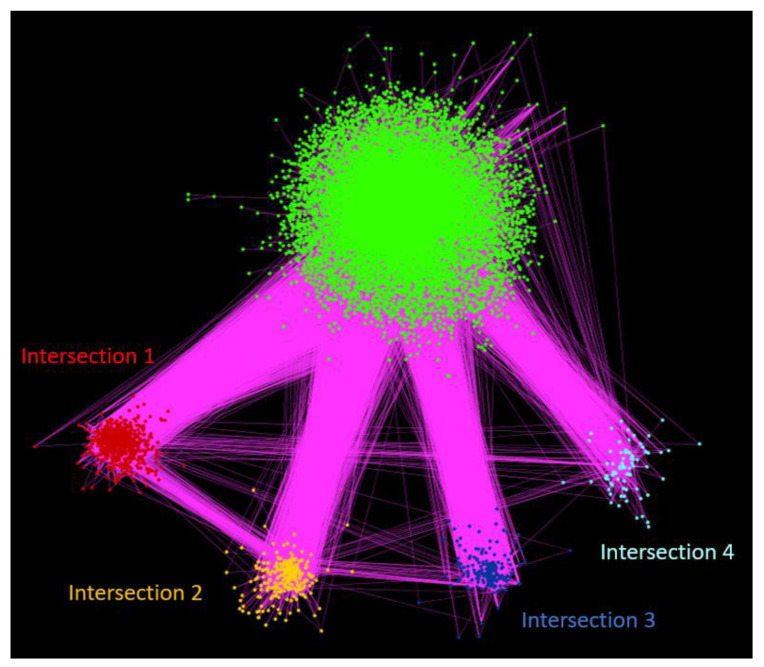
The four largest intersections between clusters of the two clustering methods, RW and N2V-HC, applied on the full PICKLE 3.2 PPI network. These groups of nodes represent densely connected neighborhoods of the human protein interactome, referring to concrete biological functions. Red, yellow, blue, and light blue dots represent the protein-node sets of intersections 1 (RW Cluster 5 // N2V-HC Cluster 2), 2 (RW Cluster 8 // N2V-HC Cluster 3), 3 (RW Cluster 10 // N2V-HC Cluster 8), and 4 (RW Cluster 13 // N2V-HC Cluster 10), respectively, ranked by size. Green dots represent the rest of the protein-nodes of PICKLE 3.2 interactome. Full protein list for the four intersections is provided in Supplementary Table S7.

We performed clustering analysis based on two algorithms as described in Materials and Methods, to identify closely connected neighborhoods and investigate whether these coincide with certain biological processes, molecular functions and/or cellular components, by extracting biological insight from the full network. The clusters to which each human PPI network protein belongs for both algorithms are provided in Supplementary Table S7 for both clustering methods; the UniProt/SwissProt PE level of each protein is also shown. Supplementary Table S8 depicts the correlation matrix between the 15 largest clusters of both algorithms. The RW algorithm produced four large clusters with over 1000 members and then the cluster size dropped quickly. The N2V-HC provided two large clusters with more than 1000 members, while the rest of the clusters presented a smoother size distribution. As the two algorithms provide a different perspective of the network connectivity, we focused our analysis on four protein neighborhoods that were identified by both algorithms, as indicated by the high overlap between their respective clusters (Supplementary Table S8). The high connectivity of these neighborhoods was further investigated in the context of the biological role of the involved proteins and the biological functions to which they belong, further supporting the usefulness of the holistic analysis of the PPI network to extract biologically relevant information. Figure 4 shows the structure of these subnetworks as extracted from the full human protein interaction network. More specifically, the four largest intersections are as follows; the proteins belonging to each intersection can be found in Supplementary Table S7.

#### 3.4.1. Intersection 1: Between RW Cluster 5 and N2V-HC Cluster 2

It is the largest common cluster including 685 proteins, which are mainly characterized as an “Integral component of membrane” related to “transmembrane transport”. The major identified subgroups are related to the “endoplasmic reticulum membrane” and “Golgi membrane”. Fractions of multiprotein complexes that are parts of this intersection, include:(a) five proteins of the urea transmembrane transport activity), (b) four proteins of the L-lysine and arginine transmembrane transporter activities), (c) the connexon complex (nine proteins), involved in forming the pore of a gap junction between adjacent cells, and associated with diabetes, cardiovascular disease and cancer, and, (d) 12 members of the claudin protein family, significant components of the tight junctions.

#### 3.4.2. Intersection 2: Between RW Cluster 8 and N2V-HC Cluster 3

**Figure 5:**
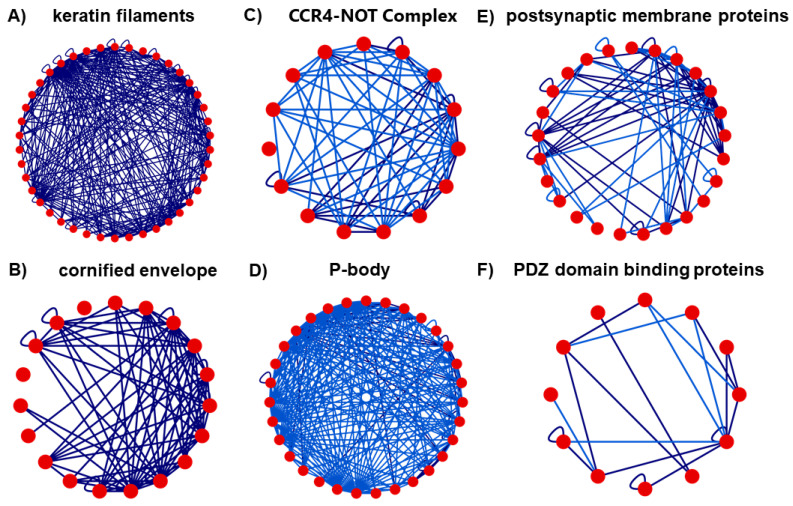
Multiprotein complexes with distinct biological functionality extracted from cluster intersections 2, 3 and 4: Keratin filaments (**A**) and cornified envelope (**B**) protein complexes of intersection 2; CCR-4 NOT (**C**) and P-body (**D**) protein complexes of intersection 3; Postsynaptic membrane (**E**) and PDZ domain binding proteins (**F**) protein complexes of intersection 4. Only the PPIs between the proteins of these complexes in the cluster intersections are shown, as provided and visualized in PICKLE website.

It includes 245 proteins. Functional analysis indicated enrichment in keratin filament (41 proteins), which anchors the skin cells to the extracellular matrix, and cornified envelope (19 proteins), building a protecting barrier of human skin against the environment (Figure 5A,B, respectively).

#### 3.4.3. Intersection 3: Between RW Cluster 10 and N2V-HC Cluster 8

It includes 177 proteins. Functional analysis indicated enrichment in proteins related to the poly (A) RNA binding (104 proteins in total), the CCR4-NOT complex (15 proteins) involved in the regulation of mRNA metabolism (Figure 5C) and the cytoplasmic mRNA processing P-body (31 proteins) involved in post-transcriptional regulation (Figure 5D).

#### 3.4.4. Intersection 4: Between RW Cluster 13 and N2V-HC Cluster 10

It includes 76 proteins. Functional analysis indicated enrichment in proteins related to (a) potassium ion transport (10 proteins), postsynaptic membrane (25 proteins) (Figure 5E) and PDZ domain binding (12 proteins) involved in assembling signaling complexes and localizing enzymes with their substrates [36] (Figure 5F).

## 4. Discussion

### 4.1. A Structurally Defined Reconstruction of the Human Protein Interactome Has Now Been Reached

In this study, we analyzed the successive releases of the PICKLE PPI meta-database from 2013 to 2021 to evaluate the global and the local expansion of the human protein interactome and attempt to give an answer on how close the scientific community is to completing the genome-scale reconstruction of the human protein interactome. Our observations support the argument that an almost complete picture of a structurally defined human protein interactome has now been reached. The human direct PPI network has recently been enriched, mainly in PPIs and not in protein nodes, having reached a coverage of at most 88% of the reviewed human complete proteome (RHCP) in its unfiltered form. The new PPIs are mainly derived from high-throughput experiments, while the vast majority of the newly added protein nodes have a low number of interactions. The hubs of the network have been largely identified, and new, if any, hubs are related to specific functions and/or tissues and are determined based on specialized experiments. Such cases are the Amyloid-beta A4 protein (UniProt ID P05067), which had 124 interactions in PICKLE 1.0 and over 2000 in subsequent versions with this increase attributed to one study [37] and Huntingtin (UniProt ID P42858), which showed an increment from 248 in PICKLE 2.6 to 903 PPIs in PICKLE 3.2, based on a 2020 study [32]. The conclusion about an almost complete reconstruction of the human protein interaction network is also supported by a largely overlapping k-core, the most connected part of the network, between the various interactome reconstructions. New experiments enrich the network with new PPIs between existing protein nodes and it was observed that, in this way, previously remote protein neighborhoods become connected, thus decreasing the network diameter and the number of independent connected subnetworks. We envisage that the human protein interactome may be substantially enriched by the development and application of advanced methodologies including (i) the highly sensitive quantitative FRET technology for the determination of PPI affinity in high-throughput assays, especially for proteins that are difficult to be expressed and for PPIs in living cells [38]; (ii) the functional protein microarrays, especially for the quantitation of PPIs of membrane proteins for receptor interactions [39,40]; and (iii) the application and evaluation of advanced, more quantitative mass spectrometry technologies [41]. These technologies could assist in the validation of the currently identified PPIs as their vast majority is presently supported by a single publication. Consistently combining proteomic with localization, imaging and other biological (including PPI) data, as undertaken by NeXtProt and the HUPO HPP, could also substantially contribute to determine erroneous PPIs.

### 4.2. The Fraction of RHCP Proteins without Identified Interactions

The human protein interactome in 2013, as reconstructed in PICKLE 1.0, covered ~60% of RHCP, indicating that 40% of the RHCP proteins had no known experimentally determined PPI at that time, mostly glycoproteins. As the interactome evolved over the years, the number of RHCP proteins with no interactions has substantially decreased, reaching 18% when considering the cross-checked human interactome reconstruction, the strictest with respect to the probability for an experimentally determined PPI to be direct. This number is further decreased to 12% of RHCP in the unfiltered PICKLE reconstruction of the human protein interactome, which includes all experimentally determined PPIs independently of their reliability score of being direct. This small fraction, which has been rather stable in the last three releases of PICKLE, further supports the structural completeness of the interactome. Our analysis of these proteins with respect to their protein evidence level indicated that ~20% of the proteins with no PPIs are of an uncertain characterization. This means that the uncertain proteins can partially justify the still missing information about PPIs for this part of RHCP; there are still well-annotated proteins that have no known PPIs and further investigation is required. Furthermore, the nature of these proteins, which are largely enriched in olfactory receptors and their coupled G-proteins, justifies the difficulty in identifying any potential interactions, as targeted experiments may be required. Regarding the PE5 (uncertain) RHCP UniProt IDs, there is a high probability that most do not correspond to actual proteins and will become obsolete in subsequent versions of RHCP. Recently, Abascal et al. [42] supported that about 10% of the annotated genes in major databases (Ensembl/GENCODE, RefSeq and UniProtKB) may be non-coding or pseudogenes, since they present relevant features. This seems to be the case for several olfactory receptors [43]. However, our analysis indicated a smaller than 1% fraction of the protein nodes of the PICKLE human interactome to be of uncertain (PE5) evidence. Furthermore, in our data there are 60 interactions involving 83 proteins encoded by LINC genes (long intergenic non-protein coding RNA), 67 of which are annotated as “uncertain”. A fraction of this dataset is expected to be updated into fully characterized LINC-encoded microproteins [44]. In the most recent protein evidence update of NeXtProt [20], 1, 12 and 8 of the 83 uncertain protein products of LINC genes were, respectively, upgraded to PE1, PE2 and PE4 levels. Overall, these results imply that the present reconstruction of the human protein interactome may indeed be covering a higher than 90% part of RHCP than the current annotation is showing.

### 4.3. Exploring the Full Human Protein Interactome Topology Can Lead to Useful Biological Insights

A well-reconstructed human protein interactome is a powerful tool in network biology and medicine research forming the basis for multi-omic and dynamic analyses. We expect that the topology of the network and the connectivity between the various parts of the network reflect the relationship between certain biological processes or densely connected multi-protein complexes of biological relevance. Thus, the study of the connectivity of the human protein interaction network could provide useful biological insights. Based on this hypothesis and considering that the PICKLE 3.2 human PPI network reconstruction is a well-defined representation of the full human interactome, we proceeded in cross-applying two clustering algorithms to determine the protein neighborhoods that stand out due to their higher degree of connectivity. Indeed, we were able to identify four such regions of the network, which are of biological relevance as described below.

The first largest intersection consists mainly of proteins with one or more parts of their amino acid sequence embedded in the lipophilic part of cellular membrane(s), characterized as an “integral component of membrane” related to “transmembrane transport”. Within this cluster, several protein groups are identified, including five members of the urea transmembrane transport activity involved in urine concentration and the regulation of renal water excretion, with the L-lysine and arginine transmembrane transporter activities playing a significant role in macrophage activation and proliferation [45]; nine proteins of the connexon complexes forming connexin hemichannels allowing for the bidirectional flow of ions and playing a key role in intracellular signaling [46]; and twelve members of the claudin protein family involved in calcium-independent via plasma membrane cell–cell adhesion associated with inflammation, cancer and metabolic disorder [47].

In the second largest intersection, two large group of proteins were found, related with keratin intermediate filaments (Figure 5A) and the cornified envelope (Figure 5B). Keratin intermediate filaments are part of the epithelial cytoskeleton, in combination with microtubules and actin filaments. They are abundant in the terminally differentiated keratinocytes of the epidermis cornified envelope, a robust protein/lipid structure, which consists of proteins (e.g., LCE3A, LCE3B. LCE3C) involved in an innate cutaneous host defense exhibiting defensin-like anti-microbial activity [48]. It has been suggested that genes of the late cornified envelope 3 (LCE-3) gene family may be associated with psoriasis and psoriatic arthritis, and may also have a pleiotropic effect on some autoimmune diseases [49].

The protein set in the third intersection is enriched in poly (A)-RNA binding (PABPs) proteins, which are involved in post-transcriptional modifications of poly (A)+ mRNA molecules and control their function. In the nucleus, this major group of regulatory factors contribute to the synthesis and final length of the poly (A) tail, in the maturation of mRNA molecules and their export to the cytoplasm, where they promote translation initiation and termination, mRNA stability and ribosome recycling. Among PABPs, the multiprotein CCR4-NOT complex (Figure 5C) is associated with the efficiency of translation being involved in poly (A) tail shortening [50], and the cytoplasmic mRNA processing body (P-body) (Figure 5D), which participates in mRNA decay [51].

Finally, the fourth and smallest among these four intersections is characteristically enriched in: (a) 10 proteins of the potassium voltage-gated channel subfamilies A and J playing important roles in the biology of the central nervous system. Among these is ATP-sensitive inward rectifier potassium channel 10 protein, encoded by *KCNJ10*, potentially responsible for the potassium buffering action of brain glial cells. Variants of this protein cause SESAME syndrome, a complex neurological disease; (b) 25 highly clustered postsynaptic membrane proteins (Figure 5E), including dystrophin (UniProt ID: P11532), a component of the dystrophin-associated glycoprotein complex localized at a variety of synapses in the nervous system and the neuromuscular junction, encoded by *DMD*, the causal gene of Duchenne muscular dystrophy, and Neuroligin-4 (UniProt ID: Q8N0W4) encoded by *NLGN4X*, associated with X-linked autism and Asperger syndrome [52]; (c) 12 highly interconnected PDZ domain proteins (Figure 5F). These proteins are, in general, involved in the surface retention of various ion channels and cellular trafficking and have been connected to several neurological disorders, cancer and cystic fibrosis [53]. In particular, dystrobrevin alpha, encoded by *DTNA*, is causally involved in Barth syndrome, a severe infantile cardiomyopathy [54], and Frizzled-4 protein, a receptor for Wnt proteins, encoded by *FZD4*, is causally involved in familial exudative vitreoretinopathy leading to an avascular peripheral retina [55]. Interestingly, the three protein groups are physically and functionally intersecting through a PDZ domain protein encoded by *KCNJ4,* named Inward rectifier potassium channel 4 protein (UniProt ID: P48050), which is involved in potassium ion import across the plasma membrane. In addition, four other PDZ domain proteins of cluster (c), i.e., the Disks large homolog 3 (UniProt ID: Q92796) encoded by *DLG3*, involved in the regulation of postsynaptic membrane neurotransmitter receptor levels; the Disks large homolog 4 protein (UniProt ID: P78352) encoded by *DLG4*, a postsynaptic scaffolding protein playing a significant role in synaptogenesis and synaptic plasticity; and, finally, the Protein lin-7 homolog B (UniProt ID: Q9HAP6) encoded by *LIN7B* and the Protein lin-7 homolog C (UniProt ID: Q9NUP9) encoded by *LIN7C*, both of which are involved, among other functions, in neurotransmitter secretion, are also members of the postsynaptic membrane proteins group of (b).

## Data Availability

The datasets from various PICKLE releases are available for download at http://www.pickle.gr (accessed on 10 January 2022); all other results are provided in Supplementary Materials.

## Supplementary Materials

The following are available online at https://www.mdpi.com/article/10.3390/biom12010140/s1, Table S1: The human protein interactome expansion over successive PICKLE releases and the contribution of source databases in terms of UniProt IDs, PPIs and supporting publications based on the default (cross-checked) reconstruction, Table S2: The human protein interactome expansion over successive PICKLE releases in terms of UniProt IDs, PPIs and supporting publications in the three filtering modes (cross-checked (default), standard and unfiltered), Table S3: Distribution of RHCP UniProt IDs in all PICKLE releases according to experimental evidence as reported by UniProt and NextProt, Table S4: A. The list of the RHCP UniProt IDs without any PPI in all PICKLE releases and their UniProt and NeXtProt experimental evidence; B. Distribution of UniProt IDs without PPIs according to their UniProt and NeXtProt experimental evidence in all PICKLE releases, Table S5: Metrics of the human PPI network in all PICKLE releases, Table S6: A. The degree of each UniProt ID in the human protein interactome in all PICKLE releases; B. The distribution of UniProt IDs in the PPI network according to the UniProt experimental evidence in all PICKLE releases, Table S7: Assignment of the PICKLE 3.2 human protein interactome proteins to clusters based on the RW and N2V-HC algorithms., Table S8: The correlation matrix between the 15 largest RW and N2V-HC clusters, Figure S1: The distribution of (A) the number of supporting publications per PPI and (B) the number of PPIs reported in a reference in PICKLE 3.2, Figure S2: The number (A) and the percentage (B) of RHCP proteins with and without PPIs in all PICKLE releases.
