# Supplementary material for: How Far Are We from the Completion of the Human Protein Interactome Reconstruction?
Source: Biomolecules. 2022 Jan 15;12(1):140. doi: 10.3390/biom12010140 (PMC8774112; doi:10.3390/biom12010140)
Supplement: Supplementary file 1 [file biomolecules-12-00140-s001.zip › biomolecules-1523777 proof supplementary/Supplementary_Material.pdf]

## Supplementary Material

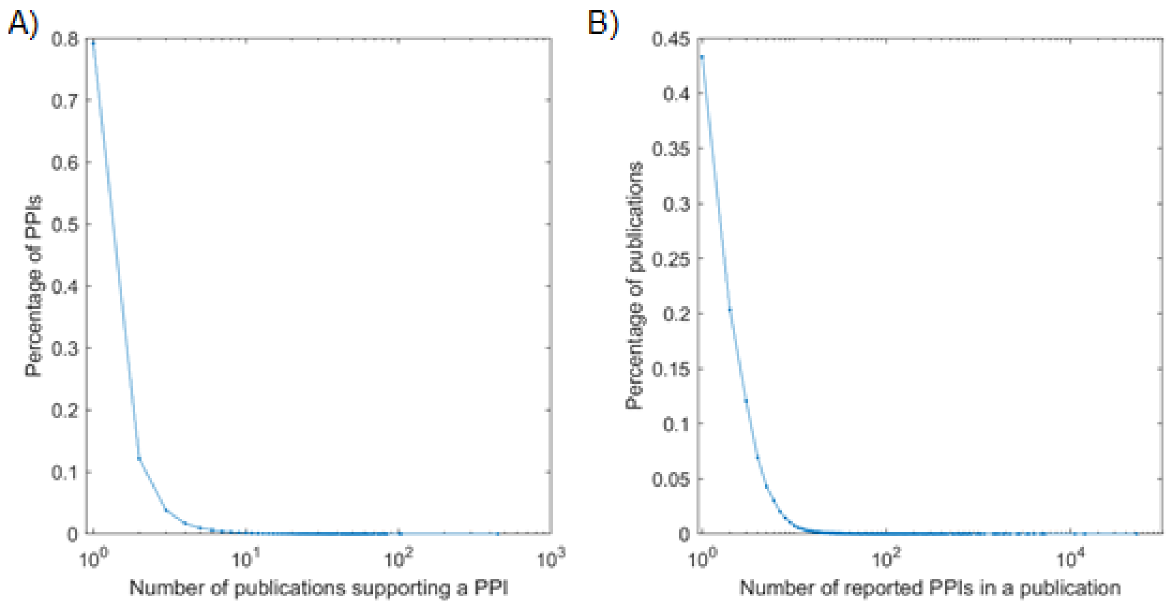


**Figure S1. The distribution of (A) the number of supporting publications per PPI and (B) the number of PPIs reported in a reference in PICKLE 3.2.**

**Table S1. The human protein interactome expansion over successive PICKLE releases and the contribution of source databases in terms of UniProt IDs, PPIs and supporting publications based on the default (cross-checked) reconstruction.**

| | UniProt IDs | | | | | | | |
|---|---|---|---|---|---|---|---|---|
| | PICKLE 1.0 | PICKLE 2.1 | PICKLE 2.2 | PICKLE 2.3 | PICKLE 2.4 | PICKLE 2.5 | PICKLE 2.6 | PICKLE 3.2 |
| HPRD | 9303 | 9358 | 9343 | 9355 | 9357 | 9358 | 9378 | 9385 |
| IntAct | 6666 | 8958 | 12571 | 13193 | 14261 | 14274 | 15275 | 14912 |
| BioGRID | 9265 | 12890 | 13277 | 13447 | 13588 | 13613 | 15131 | 15361 |
| DIP | 1795 | 2686 | 3135 | 3136 | 3136 | 3133 | | |
| MINT | 6102 | 5134 | 5360 | 5368 | 5811 | 5826 | | |
| **PICKLE** | **11827** | **14134** | **15434** | **15823** | **16418** | **16418** | **16568** | **16384** |
| RHCP | 20242 | 20193 | 20230 | 20381 | 20418 | 20350 | 20375 | 20394 |

| | PPIs | | | | | | | |
|---|---|---|---|---|---|---|---|---|
| | PICKLE 1.0 | PICKLE 2.1 | PICKLE 2.2 | PICKLE 2.3 | PICKLE 2.4 | PICKLE 2.5 | PICKLE 2.6 | PICKLE 3.2 |
| HPRD | 37152 | 37313 | 37450 | 37542 | 37550 | 37538 | 37680 | 37781 |
| IntAct | 19425 | 29536 | 75156 | 88935 | 95807 | 95806 | 123635 | 138186 |
| BioGRID | 42647 | 90452 | 96747 | 100768 | 106057 | 107593 | 161433 | 171377 |
| DIP | 2609 | 4811 | 5932 | 5934 | 5934 | 5929 | | |
| MINT | 16147 | 11667 | 13004 | 12998 | 14050 | 14333 | | |
| **PICKLE** | **75965** | **120882** | **161007** | **178306** | **189693** | **191113** | **201524** | **214446** |

| | References | | | | | | | |
|---|---|---|---|---|---|---|---|---|
| | PICKLE 1.0 | PICKLE 2.1 | PICKLE 2.2 | PICKLE 2.3 | PICKLE 2.4 | PICKLE 2.5 | PICKLE 2.6 | PICKLE 3.2 |
| HPRD | 19267 | 19484 | 19490 | 19519 | 19522 | 19519 | 19513 | 19524 |
| IntAct | 1598 | 2007 | 2396 | 2687 | 2752 | 2891 | 9506 | 9624 |
| BioGRID | 13818 | 23305 | 25179 | 26342 | 27938 | 28847 | 30263 | 30507 |
| DIP | 1180 | 2094 | 2430 | 2431 | 2431 | 2431 | | |
| MINT | 2398 | 2831 | 2897 | 2975 | 2996 | 2997 | | |
| **PICKLE** | **26689** | **35752** | **38522** | **39603** | **41170** | **42121** | **43310** | **44634** |

**Notes**:

- PICKLE integrates the primary PPI datasets based on the reviewed human complete proteome (RHCP) genetic information network;
- In PICKLE 1.0, all primary datasets are mined from their own resource.
- From PICKLE 2.1 to PICKLE 2.5, the IntAct dataset mined included only the IntAct annotated PPIs in the IntAct - curated human dataset; the MINT dataset was mined from the MINT-annotated PPIs of the IntAct-curated human dataset; DIP PPIs were mined from its own resource.
- Since PICKLE 2.6, the entired dataset of IntAct is curated into PICKLE, including the MINT and DIP annotated PPIs and other primary PPI datasets
- The PICKLE 1.0 dataset refers to the standard reconstruction of the subsequent PICKLE releases.

**Table S2. The human protein interactome expansion over successive PICKLE releases in terms of UniProt IDs, PPIs and supporting publications in the three filtering modes (cross-checked (default), standard, and unfiltered).**

| | | **PICKLE 1.0** | **PICKLE 2.1** | **PICKLE 2.2** | **PICKLE 2.3** | **PICKLE 2.4** | **PICKLE 2.5** | **PICKLE 2.6** | **PICKLE 3.2** |
|---|---|---|---|---|---|---|---|---|---|
| Release date | | Jan. 2012 | Aug. 2017 | May 2018 | Oct. 2018 | Jun. 2019 | Nov. 2019 | Sep. 2020 | Mar. 2021 |
| UniProt Version | | 2011_12 | 2015_02 | 2017_12 | 2018_09 | 2019_05 | 2019_10 | 2020_03 | 2020_06 |
| RHCP | | 20242 | 20193 | 20230 | 20381 | 20416 | 20350 | 20375 | 20394 |
| UniProt IDs | Cross-checked | ND | 14134 | 15434 | 15823 | 16418 | 16418 | 16568 | 16384 |
| | Standard | 11827 | 14193 | 15483 | 15868 | 16456 | 16560 | 16703 | 16803 |
| | Unfiltered | ND | 15725 | 17064 | 17300 | 17694 | 17706 | 17824 | 17915 |
| PPIs | Cross-checked | ND | 120882 | 161007 | 178306 | 189693 | 191113 | 201524 | 214446 |
| | Standard | 75965 | 124871 | 165568 | 182921 | 194346 | 196511 | 207456 | 224842 |
| | Unfiltered | ND | 191647 | 334069 | 367709 | 409868 | 425340 | 475056 | 528710 |
| References | Cross-checked | ND | 35752 | 38522 | 39603 | 41170 | 42121 | 44310 | 44634 |
| | Standard | 26689 | 35774 | 38474 | 39609 | 41176 | 42127 | 44321 | 44645 |
| | Unfiltered | ND | 35930 | 38469 | 39664 | 41231 | 42189 | 44419 | 44741 |

**Notes**:

- PICKLE integrates the primary PPI datasets based on the reviewed human complete proteome (RHCP) genetic information network;
- In PICKLE 1.0, all primary datasets are mined from their own resource.
- From PICKLE 2.1 to PICKLE 2.5, the IntAct dataset mined included only the IntAct annotated PPIs in the IntAct - curated human dataset; the MINT dataset was mined from the MINT-annotated PPIs of the IntAct-curated human dataset; DIP PPIs were mined from its own resource.
- Since PICKLE 2.6, the entired dataset of IntAct is curated into PICKLE, including the MINT and DIP annotated PPIs and other primary PPI datasets
- The PICKLE 1.0 dataset refers to the standard reconstruction of the subsequent PICKLE releases.

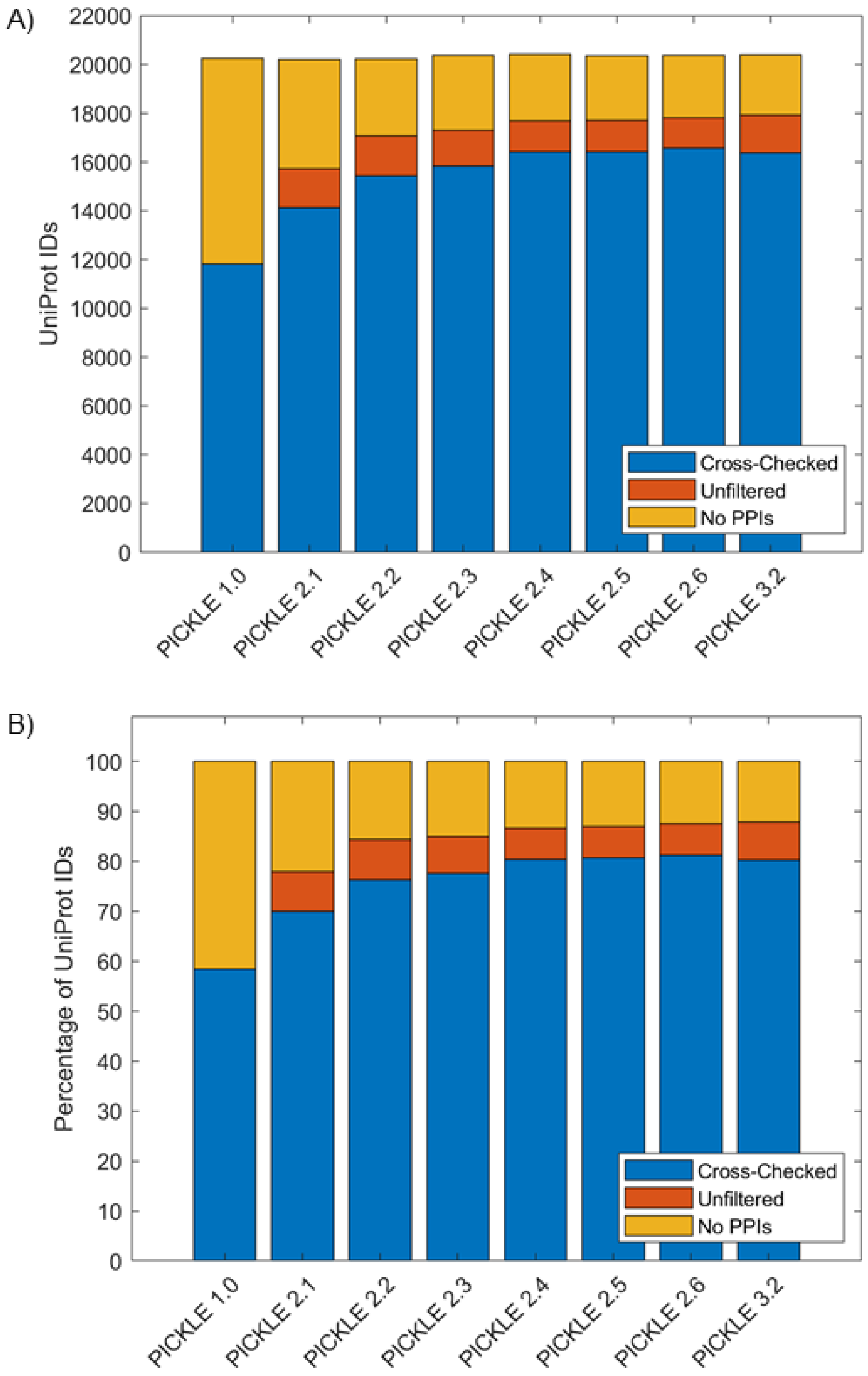


**Figure S2. The number (A) and the percentage (B) of RHCP nodes with and without PPIs in all PICKLE releases.** The RHCP proteins in the default (cross-checked) PICKLE network are shown in blue; the proteins having PPIs only in unfiltered network are shown in orange; the RHCP proteins without PPIs are shown in yellow. All PICKLE releases refer to an almost constant RHCP (only 1% difference between various datasets).

**Table S3. Distribution of RHCP UniProt IDs in all PICKLE releases according to experimental evidence as reported by UniProt and NextProt.** The NeXtProt datasets were selected to be chronologically relevant to the UniProt versions used in PICKLE releases.

| | **PICKLE 2.1** | | **PICKLE 2.2** | | **PICKLE 2.3** | | **PICKLE 2.4** | | **PICKLE 2.5** | | **PICKLE 2.6** | | **PICKLE 3.2** | |
|---|---|---|---|---|---|---|---|---|---|---|---|---|---|---|
| **Protein Evidence Level** | **UniProt** | **NeXtProt** | **UniProt** | **NeXtProt** | **UniProt** | **NeXtProt** | **UniProt** | **NeXtProt** | **UniProt** | **NeXtProt** | **UniProt** | **NeXtProt** | **UniProt** | **NeXtProt** |
| PE1 | 15383 | 16684 | 15314 | 17470 | 15293 | 17487 | 15412 | 17690 | 15411 | 17623 | 16311 | 18260 | 16373 | 18258 |
| PE2 | 3835 | 2141 | 3501 | 1660 | 3567 | 1728 | 3454 | 1544 | 3388 | 1542 | 2555 | 1186 | 2522 | 1185 |
| PE3 | 284 | 534 | 702 | 452 | 799 | 515 | 811 | 508 | 814 | 506 | 757 | 248 | 757 | 246 |
| PE4 | 86 | 93 | 139 | 74 | 151 | 76 | 161 | 71 | 296 | 71 | 141 | 44 | 141 | 44 |
| PE5 | 605 | 596 | 574 | 574 | 571 | 571 | 578 | 570 | 441 | 569 | 611 | 608 | 601 | 606 |
| **RHCP UniProt IDs not in NeXtProt** | | **145** | | | | **4** | | **33** | | **39** | | **29** | | **55** |
| TOTAL | 20193 | | 20230 | | 20381 | | 20416 | | 20350 | | 20375 | | 20394 | |

PE1. Experimental evidence at protein level
PE2. Experimental evidence at transcript level
PE3. Protein inferred from homology
PE4. Protein predicted
PE5. Protein uncertain

**Table S5. Metrics of the human PPI network in all PICKLE releases.**

| Network Characteristic | PICKLE 1.0 | PICKLE 2.1 | PICKLE 2.2 | PICKLE 2.3 | PICKLE 2.4 | PICKLE 2.5 | PICKLE 2.6 | PICKLE 3.2 |
|---|---|---|---|---|---|---|---|---|
| Number of Nodes | 11827 | 14134 | 15434 | 15823 | 16418 | 16418 | 16568 | 16384 |
| Isolated Nodes (homodimers) | 114 | 68 | 51 | 43 | 23 | 34 | 33 | 39 |
| Connected components | 174 (1 cluster of 11577 nodes, 114 homodimers, 46 heterodimers, 13 isolated components of 3 or 4 nodes) | 95 (1 cluster of 14010 nodes, 68 homodimers, 22 heterodimers, 4 three-node isolated components) | 66 (1 cluster of 15352 nodes, 51 homodimers, 11 heterodimers, 3 three-node isolated components) | 55 (1 cluster of 15756 nodes, 43 homodimers, 9 heterodimers, 2 three-node isolated components) | 42 (1 cluster of 16368 nodes, 33 homodimers, 7 heterodimers, 1 three-node isolated component) | 42 (1 cluster of 16369 nodes, 34 homodimers, 6 heterodimers, 1 three-node isolated component) | 42 (1 cluster of 16518 nodes, 33 homodimers, 7 heterodimers, 1 three-node isolated component) | 50 (1 cluster of 16323 nodes, 39 homodimers, 8 heterodimers, 2 three-node isolated component) |
| Number of self-loops | 2715 (i.e.: 2601 nodes having interactions with other proteins as well, and 114 isolated homodimers) | 3515 (i.e.: 3447 nodes having interactions with other proteins as well, and 68 isolated homodimers) | 3794 (i.e.: 3743 nodes having interactions with other proteins as well, and 51 isolated homodimers) | 3879 (i.e.: 3836 nodes having interactions with other proteins as well, and 43 isolated homodimers) | 3940 (i.e.: 3907 nodes having interactions with other proteins as well, and 33 isolated homodimers) | 3966 (i.e.: 3934 nodes having interactions with other proteins as well, and 34 isolated homodimers) | 4119 (i.e.: 3986 nodes having interactions with other proteins as well, and 33 isolated homodimers) | 4135 (i.e.: 3996 nodes having interactions with other proteins as well, and 39 isolated homodimers) |
| Network diameter | 12 | 10 | 9 | 9 | 9 | 8 | 8 | 8 |
| Characteristic Path Length | 3.691 | 3.35 | 3.342 | 3.315 | 3.304 | 3.297 | 3.274 | 3.215 |
| Average Number of Neighbors | 12.387 | 16.608 | 20.372 | 22.047 | 22.628 | 22.798 | 23.83 | 25.673 |
| Shortest Paths | 95% | 98% | 98% | 99% | 99% | 99% | 99% | 99% |
| Clustering Coefficient | 0.127 | 0.134 | 0.121 | 0.116 | 0.109 | 0.109 | 0.108 | 0.113 |
| Network Density | 0.001 | 0.001 | 0.001 | 0.001 | 0.001 | 0.001 | 0.001 | 0.002 |
| Network Centralization | 0.093 | 0.153 | 0.133 | 0.13 | 0.128 | 0.129 | 0.129 | 0.143 |
| Network Heterogeneity | 2.193 | 2.567 | 2.102 | 2.001 | 2.001 | 2.003 | 1.97 | 1.974 |

**Table S8. The correlation matrix between the 15 largest RW and N2V-HC clusters.**

| | N2V-HC | # 1 | # 2 | # 3 | # 4 | # 5 | # 6 | # 7 | # 8 | # 9 | # 10 | # 11 | # 12 | # 13 | # 14 | # 15 |
|---|---|---|---|---|---|---|---|---|---|---|---|---|---|---|---|---|
| **RW** | **size** | **1192** | **1001** | **364** | **347** | **294** | **268** | **251** | **251** | **182** | **171** | **170** | **162** | **162** | **150** | **143** |
| **# 1** | **3932** | 9.6% | 1.2% | 0.6% | 8.6% | 1.8% | 5.3% | 12.3% | 1.6% | 1.7% | 2.8% | 3.4% | 1.5% | 11.9% | 15.0% | 2.8% |
| **# 2** | **1497** | 34.2% | 0.5% | 3.8% | 1.1% | 24.1% | 0.6% | 0.7% | 0.3% | 13.6% | 0.2% | 0.6% | 1.4% | 0.2% | 1.3% | 0.7% |
| **# 3** | **1341** | 2.9% | | | 0.2% | 0.8% | 1.8% | 4.7% | 1.3% | 0.4% | 0.2% | 17.6% | 0.4% | 2.4% | 1.3% | 0.7% |
| **# 4** | **1268** | 8.8% | 0.4% | 0.4% | 0.8% | 3.8% | 2.4% | 1.4% | 0.7% | 2.8% | 0.9% | 2.6% | 1.3% | 0.7% | 0.5% | 1.2% |
| **# 5** | **827** | 0.2% | **75.3%** | 0.6% | 0.2% | 0.2% | 0.6% | 0.4% | 0.2% | | | | 1.6% | | | 0.6% |
| **# 6** | **501** | 1.9% | 9.6% | 1.2% | 2.9% | 1.4% | 1.9% | 1.7% | 0.3% | 1.7% | 0.3% | 0.3% | 4.6% | 0.4% | 0.7% | |
| **# 7** | **479** | 1.3% | | | | 1.9% | 1.7% | 0.9% | 2.2% | 0.7% | 0.4% | 0.4% | | 5.7% | 0.4% | |
| **# 8** | **386** | 1.5% | 0.3% | **65.6%** | 0.3% | 7.4% | | | 0.6% | 1.6% | 0.8% | | 1.2% | | | |
| **# 9** | **375** | 0.8% | 0.5% | | 39.6% | 0.3% | 1.0% | | | 0.4% | | 0.4% | 0.8% | 0.4% | | |
| **# 10** | **255** | 0.7% | | | | | | 0.4% | **70.0%** | | | | | | | 0.5% |
| **# 11** | **171** | 1.3% | 0.7% | 1.2% | 0.4% | 0.9% | 0.9% | | | 0.6% | 1.2% | | | | 0.6% | |
| **# 12** | **119** | 0.5% | 1.7% | | 1.0% | | 3.4% | | | | | 2.1% | | | | |
| **# 13** | **104** | 1.1% | 0.3% | | | | | 1.2% | | | **57.0%** | 1.5% | | | | 0.8% |
| **# 14** | **87** | | | | | | | | | | | | | | | **72.6%** |
| **# 15** | **82** | 0.6% | 0.4% | 0.6% | | 0.6% | 2.2% | | | | | | | | | |

Note: The correlation coefficient between two clusters is estimated by the ratio of the number of proteins in their intersection divided by their geometric mean
