# Supplementary material for: How Far Are We from the Completion of the Human Protein Interactome Reconstruction?
Source: Biomolecules. 2022 Jan 15;12(1):140. doi: 10.3390/biom12010140 (PMC8774112; doi:10.3390/biom12010140)
Supplement: Supplementary file 1 [file biomolecules-12-00140-s001.zip › DIMITRAKOPOULOS et al_Supplementary_Material.pdf]

Supplementary Materials

Table of Contents

Figure S1. The distribution of (A) the number of supporting publications per PPI and (B) the number of PPIs reported in a reference in PICKLE 3.2…………………………….............. **2**

Table S1. The human protein interactome expansion over successive PICKLE releases and the contribution of source databases in terms of UniProt IDs, PPIs and supporting publications based on the default (cross-checked) reconstruction…………………………………………. **3**

Table S2. The human protein interactome expansion over successive PICKLE releases in terms of UniProt IDs, PPIs and supporting publications in the three filtering modes (cross-checked (default), standard, and unfiltered)……………………………………………………… **4**

Figure S2. The number (A) and the percentage (B) of RHCP nodes with and without PPIs in all PICKLE releases………………………………………………………………………. **5**

Table S3. Distribution of RHCP UniProt IDs in all PICKLE releases according to experimental evidence as reported by UniProt and NextProt…………………………………………… **6**

Table S4. A. The list of the RHCP UniProt IDs without any PPI in all PICKLE releases and their UniProt and NeXtProt experimental evidence; B.Distribution of UniProt IDs without PPIs according to their UniProt and NeXtProt experimental evidence in all PICKLE releases *(provided as a separate Excel file)*

Table S5. Metrics of the human PPI network in all PICKLE releases…………………………. **7**

Table S6. A. The degree of each UniProt ID in the human protein interactome in all PICKLE releases; B. The distribution of UniProt IDs in the PPI network according to te UniProt experimental evidence in all PICKLE releases *(provided as a separate Excel file)*

Table S7. Assignment of the PICKLE 3.2 human protein interactome proteins to clusters based on the RW and N2V-HC algorithms *(provided as a separate Excel file)*

Table S8. The correlation matrix between the 15 largest RW and N2V-HC clusters……………. **8**

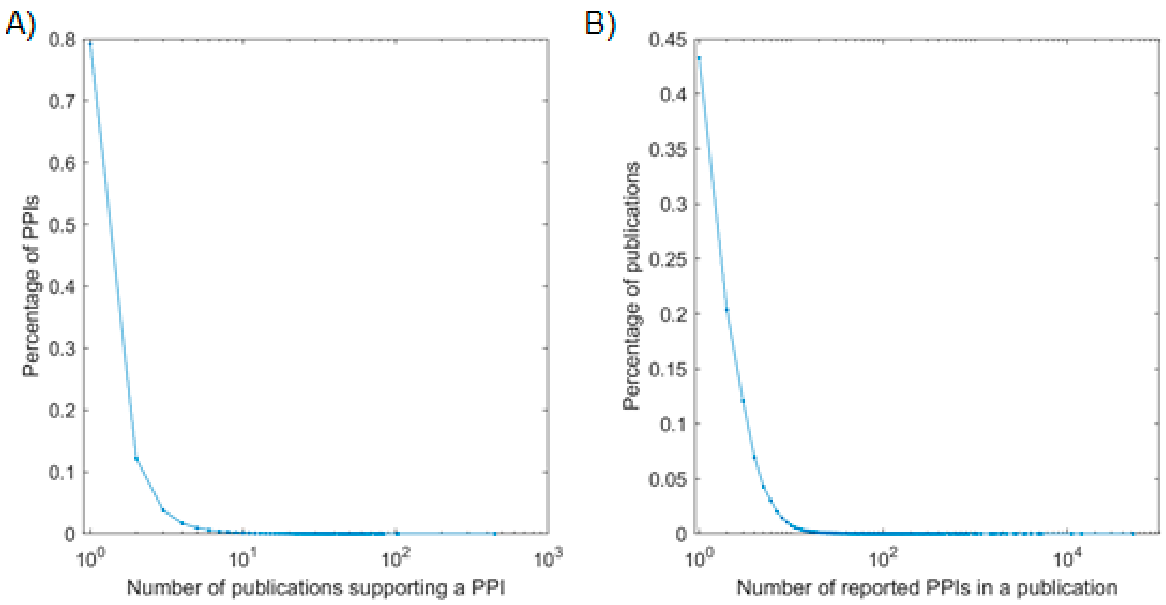

Figure S1. The distribution of (A) the number of supporting publications per PPI and (B) the number of PPIs reported in a reference in PICKLE 3.2.

Table S1. The human protein interactome expansion over successive PICKLE releases and the contribution of source databases in terms of UniProt IDs, PPIs and supporting publications based on the default (cross-checked) reconstruction.

	UniProt IDs							
	PICKLE 1.0	PICKLE 2.1	PICKLE 2.2	PICKLE 2.3	PICKLE 2.4	PICKLE 2.5	PICKLE 2.6	PICKLE 3.2
**HPRD**	9303	9358	9343	9355	9357	9358	9378	9385
**IntAct**	6666	8958	12571	13193	14261	14274	15275	14912
**BioGRID**	9265	12890	13277	13447	13588	13613	15131	15361
**DIP**	1795	2686	3135	3136	3136	3133		
**MINT**	6102	5134	5360	5368	5811	5826		
**PICKLE**	**11827**	**14134**	**15434**	**15823**	**16418**	**16418**	**16568**	**16384**
**RHCP**	20242	20193	20230	20381	20418	20350	20375	20394

	PPIs							
	PICKLE 1.0	PICKLE 2.1	PICKLE 2.2	PICKLE 2.3	PICKLE 2.4	PICKLE 2.5	PICKLE 2.6	PICKLE 3.2
**HPRD**	37152	37313	37450	37542	37550	37538	37680	37781
**IntAct**	19425	29536	75156	88935	95807	95806	123635	138186
**BioGRID**	42647	90452	96747	100768	106057	107593	161433	171377
**DIP**	2609	4811	5932	5934	5934	5929		
**MINT**	16147	11667	13004	12998	14050	14333		
**PICKLE**	**75965**	**120882**	**161007**	**178306**	**189693**	**191113**	**201524**	**214446**

	References							
	PICKLE 1.0	PICKLE 2.1	PICKLE 2.2	PICKLE 2.3	PICKLE 2.4	PICKLE 2.5	PICKLE 2.6	PICKLE 3.2
**HPRD**	19267	19484	19490	19519	19522	19519	19513	19524
**IntAct**	1598	2007	2396	2687	2752	2891	9506	9624
**BioGRID**	13818	23305	25179	26342	27938	28847	30263	30507
**DIP**	1180	2094	2430	2431	2431	2431		
**MINT**	2398	2831	2897	2975	2996	2997		
**PICKLE**	**26689**	**35752**	**38522**	**39603**	**41170**	**42121**	**43310**	**44634**

Notes:

- PICKLE integrates the primary PPI datasets based on the reviewed human complete proteome (RHCP) genetic information network;
- In PICKLE 1.0, all primary datasets are mined from their own resource.
- From PICKLE 2.1 to PICKLE 2.5, the IntAct dataset mined included only the IntAct annotated PPIs in the IntAct - curated human dataset; the MINT dataset was mined from the MINT-annotated PPIs of the IntAct-curated human dataset; DIP PPIs were mined from its own resource.
- Since PICKLE 2.6, the entired dataset of IntAct is curated into PICKLE, including the MINT and DIP annotated PPIs and other primary PPI datasets
- The PICKLE 1.0 dataset refers to the standard reconstruction of the subsequent PICKLE releases.

Table S2. The human protein interactome expansion over successive PICKLE releases in terms of UniProt IDs, PPIs and supporting publications in the three filtering modes (cross-checked (default), standard, and unfiltered).

		PICKLE 1.0	PICKLE 2.1	PICKLE 2.2	PICKLE 2.3	PICKLE 2.4	PICKLE 2.5	PICKLE 2.6	PICKLE 3.2
Release date		Jan. 2012	Aug. 2017	May 2018	Oct. 2018	Jun. 2019	Nov. 2019	Sep. 2020	Mar. 2021
UniProt Version		2011_12	2015_02	2017_12	2018_09	2019_05	2019_10	2020_03	2020_06
RHCP		20242	20193	20230	20381	20416	20350	20375	20394
UniProt IDs	Cross-checked	ND	14134	15434	15823	16418	16418	16568	16384
	Standard	11827	14193	15483	15868	16456	16560	16703	16803
	Unfiltered	ND	15725	17064	17300	17694	17706	17824	17915
PPIs	Cross-checked	ND	120882	161007	178306	189693	191113	201524	214446
	Standard	75965	124871	165568	182921	194346	196511	207456	224842
	Unfiltered	ND	191647	334069	367709	409868	425340	475056	528710
References	Cross-checked	ND	35752	38522	39603	41170	42121	44310	44634
	Standard	26689	35774	38474	39609	41176	42127	44321	44645
	Unfiltered	ND	35930	38469	39664	41231	42189	44419	44741

Notes:

- PICKLE integrates the primary PPI datasets based on the reviewed human complete proteome (RHCP) genetic information network;
- In PICKLE 1.0, all primary datasets are mined from their own resource.
- From PICKLE 2.1 to PICKLE 2.5, the IntAct dataset mined included only the IntAct annotated PPIs in the IntAct -curated human dataset; the MINT dataset was mined from the MINT-annotated PPIs of the IntAct-curated human dataset; DIP PPIs were mined from its own resource.
- Since PICKLE 2.6, the entired dataset of IntAct is curated into PICKLE, including the MINT and DIP annotated PPIs and other primary PPI datasets
- The PICKLE 1.0 dataset refers to the standard reconstruction of the subsequent PICKLE releases.

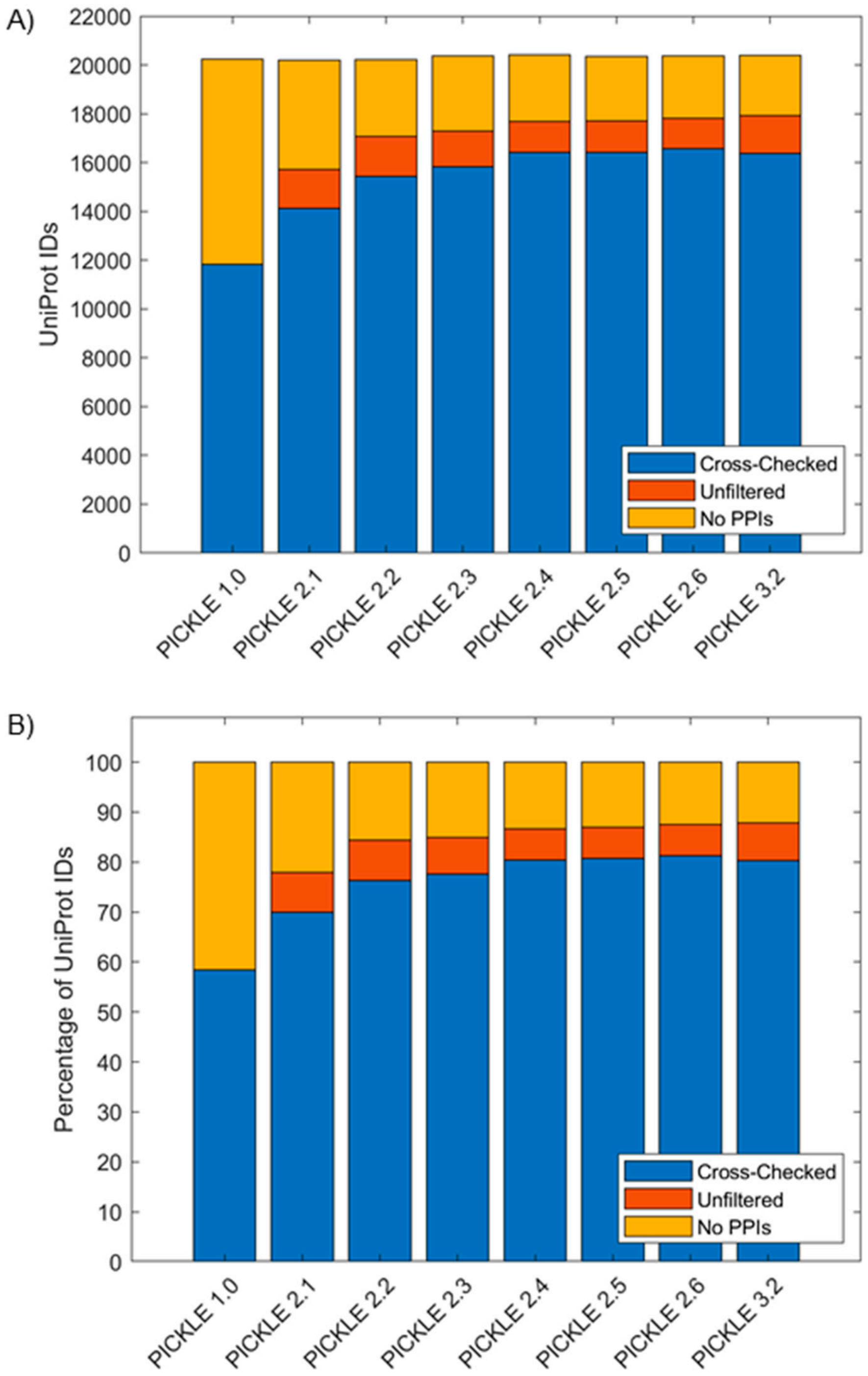

Figure S2. The number (A) and the percentage (B) of RHCP nodes with and without PPIs in all PICKLE releases. The RHCP proteins in the default (cross-checked) PICKLE network are shown in blue; the proteins having PPIs only in unfiltered network are shown in orange; the RHCP proteins without PPIs are shown in yellow. All PICKLE releases refer to an almost constant RHCP (only 1% difference between various datasets).

Table S3. Distribution of RHCP UniProt IDs in all PICKLE releases according to experimental evidence as reported by UniProt and NextProt. The NeXtProt datasets were selected to be chronologically relevant to the UniProt versions used in PICKLE releases.

	PICKLE 2.1		PICKLE 2.2		PICKLE 2.3		PICKLE 2.4		PICKLE 2.5		PICKLE 2.6		PICKLE 3.2	
**Protein Evidence Level**	**UniProt**	**NeXtProt**	**UniProt**	**NeXtProt**	**UniProt**	**NeXtProt**	**UniProt**	**NeXtProt**	**UniProt**	**NeXtProt**	**UniProt**	**NeXtProt**	**UniProt**	**NeXtProt**
PE1	15383	16684	15314	17470	15293	17487	15412	17690	15411	17623	16311	18260	16373	18258
PE2	3835	2141	3501	1660	3567	1728	3454	1544	3388	1542	2555	1186	2522	1185
PE3	284	534	702	452	799	515	811	508	814	506	757	248	757	246
PE4	86	93	139	74	151	76	161	71	296	71	141	44	141	44
PE5	605	596	574	574	571	571	578	570	441	569	611	608	601	606
**RHCP UniProt IDs not in NeXtProt**		**145**				**4**		**33**		**39**		**29**		**55**
TOTAL	20193		20230		20381		20416		20350		20375		20394	

PE1. Experimental evidence at protein level

PE2. Experimental evidence at transcript level

PE3. Protein inferred from homology

PE4. Protein predicted

PE5. Protein uncertain

Table S5. Metrics of the human PPI network in all PICKLE releases.

Network Characteristic	PICKLE 1.0	PICKLE 2.1	PICKLE 2.2	PICKLE 2.3	PICKLE 2.4	PICKLE 2.5	PICKLE 2.6	PICKLE 3.2
Number of Nodes	11827	14134	15434	15823	16418	16418	16568	16384
Isolated Nodes (homodimers)	114	68	51	43	23	34	33	39
Connected components	174 (1 cluster of 11577 nodes, 114 homodimers, 46 heterodimers, 13 isolated components of 3 or 4 nodes)	95 (1 cluster of 14010 nodes, 68 homodimers, 22 heterodimers, 4 three-node isolated components)	66 (1 cluster of 15352 nodes, 51 homodimers, 11 heterodimers, 3 three-node isolated components)	55 (1 cluster of 15756 nodes, 43 homodimers, 9 heterodimers, 2 three-node isolated components)	42 (1 cluster of 16368 nodes, 33 homodimers, 7 heterodimers, 1 three-node isolated component)	42 (1 cluster of 16369 nodes, 34 homodimers, 6 heterodimers, 1 three-node isolated component)	42 (1 cluster of 16518 nodes, 33 homodimers, 7 heterodimers, 1 three-node isolated component)	50 (1 cluster of 16323 nodes, 39 homodimers, 8 heterodimers, 2 three-node isolated component)
Number of self-loops	2715 (i.e.: 2601 nodes having interactions with other proteins as well, and 114 isolated homodimers)	3515 (i.e.: 3447 nodes having interactions with other proteins as well, and 68 isolated homodimers)	3794 (i.e.: 3743 nodes having interactions with other proteins as well, and 51 isolated homodimers)	3879 (i.e.: 3836 nodes having interactions with other proteins as well, and 43 isolated homodimers)	3940 (i.e.: 3907 nodes having interactions with other proteins as well, and 33 isolated homodimers)	3966 (i.e.: 3934 nodes having interactions with other proteins as well, and 34 isolated homodimers)	4119 (i.e.: 3986 nodes having interactions with other proteins as well, and 33 isolated homodimers)	4135 (i.e.: 3996 nodes having interactions with other proteins as well, and 39 isolated homodimers)
Network diameter	12	10	9	9	9	8	8	8
Characteristic Path Length	3.691	3.35	3.342	3.315	3.304	3.297	3.274	3.215
Average Number of Neighbors	12.387	16.608	20.372	22.047	22.628	22.798	23.83	25.673
Shortest Paths	95%	98%	98%	99%	99%	99%	99%	99%
Clustering Coefficient	0.127	0.134	0.121	0.116	0.109	0.109	0.108	0.113
Network Density	0.001	0.001	0.001	0.001	0.001	0.001	0.001	0.002
Network Centralization	0.093	0.153	0.133	0.13	0.128	0.129	0.129	0.143
Network Heterogeneity	2.193	2.567	2.102	2.001	2.001	2.003	1.97	1.974

Table S8. The correlation matrix between the 15 largest RW and N2V-HC clusters.

	N2V-HC	# 1	# 2	# 3	# 4	# 5	# 6	# 7	# 8	# 9	# 10	# 11	# 12	# 13	# 14	# 15
**RW**	**size**	**1192**	**1001**	**364**	**347**	**294**	**268**	**251**	**251**	**182**	**171**	**170**	**162**	**162**	**150**	**143**
**# 1**	**3932**	9.6%	1.2%	0.6%	8.6%	1.8%	5.3%	12.3%	1.6%	1.7%	2.8%	3.4%	1.5%	11.9%	15.0%	2.8%
**# 2**	**1497**	34.2%	0.5%	3.8%	1.1%	24.1%	0.6%	0.7%	0.3%	13.6%	0.2%	0.6%	1.4%	0.2%	1.3%	0.7%
**# 3**	**1341**	2.9%			0.2%	0.8%	1.8%	4.7%	1.3%	0.4%	0.2%	17.6%	0.4%	2.4%	1.3%	0.7%
**# 4**	**1268**	8.8%	0.4%	0.4%	0.8%	3.8%	2.4%	1.4%	0.7%	2.8%	0.9%	2.6%	1.3%	0.7%	0.5%	1.2%
**# 5**	**827**	0.2%	**75.3%**	0.6%	0.2%	0.2%	0.6%	0.4%	0.2%				1.6%			0.6%
**# 6**	**501**	1.9%	9.6%	1.2%	2.9%	1.4%	1.9%	1.7%	0.3%	1.7%	0.3%	0.3%	4.6%	0.4%	0.7%	
**# 7**	**479**	1.3%				1.9%	1.7%	0.9%	2.2%	0.7%	0.4%	0.4%		5.7%	0.4%	
**# 8**	**386**	1.5%	0.3%	**65.6%**	0.3%	7.4%			0.6%	1.6%	0.8%		1.2%			
**# 9**	**375**	0.8%	0.5%		39.6%	0.3%	1.0%			0.4%		0.4%	0.8%	0.4%		
**# 10**	**255**	0.7%						0.4%	**70.0%**							0.5%
**# 11**	**171**	1.3%	0.7%	1.2%	0.4%	0.9%	0.9%			0.6%	1.2%				0.6%	
**# 12**	**119**	0.5%	1.7%		1.0%		3.4%					2.1%				
**# 13**	**104**	1.1%	0.3%					1.2%			**57.0%**	1.5%				0.8%
**# 14**	**87**															**72.6%**
**# 15**	**82**	0.6%	0.4%	0.6%		0.6%	2.2%									

Note: The correlation coefficient between two clusters is estimated by the ratio of the number of proteins in their intersection divided by their geometric mean
